# Supplementary material for: Mosaic-PICASSO: accurate crosstalk removal for multiplex fluorescence imaging
Source: Bioinformatics. 2024 Jan 4;40(1):btad784. doi: 10.1093/bioinformatics/btad784 (PMC10781941; doi:10.1093/bioinformatics/btad784)
Supplement: btad784_Supplementary_Data [file btad784_supplementary_data.zip › Supplementary_Figures_1227.docx]

##
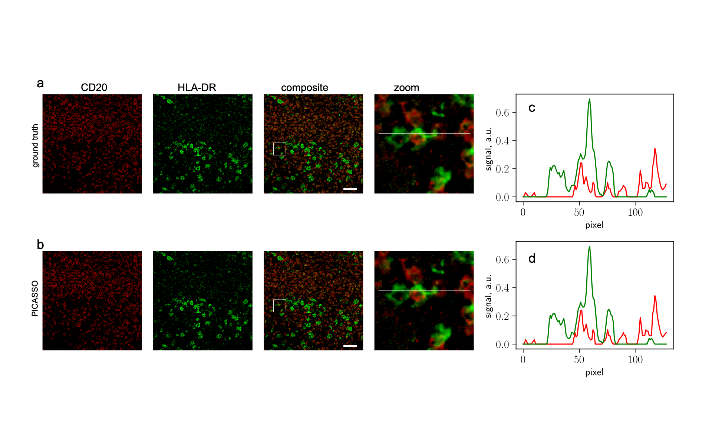
Supplementary Figures

**Supplementary Fig. 1**: (a) and (b) PICASSO correctly unmix the low overlap target pairs CD20/HLA-DR. The profiles of the ground truth (c) and PICASSO unmixing results (d) are nearly identical.


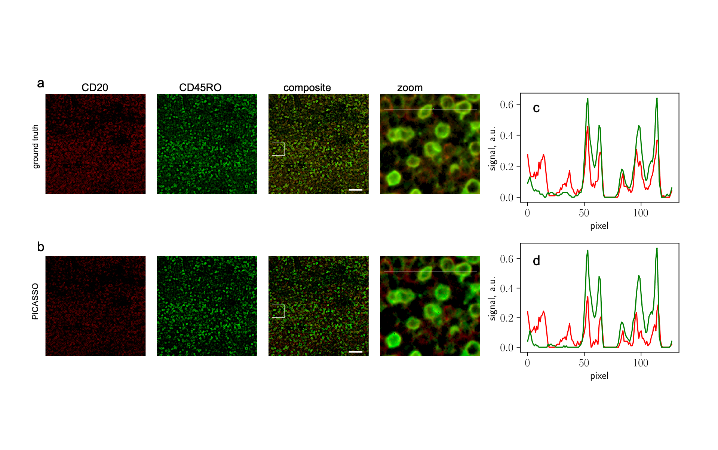


**Supplementary Fig. 2**: (a) and (b) PICASSO overcorrects for the overlap target pairs CD20/CD45RO. Comparing the profiles of the ground truth (c) and PICASSO unmixing results (d) reveals that the red channel is overly subtracted after demixing.
