# Supplementary figures and images for: Mosaic-PICASSO: accurate crosstalk removal for multiplex fluorescence imaging
Source: Bioinformatics. 2024 Jan 4;40(1):btad784. doi: 10.1093/bioinformatics/btad784 (PMC10781941; doi:10.1093/bioinformatics/btad784)

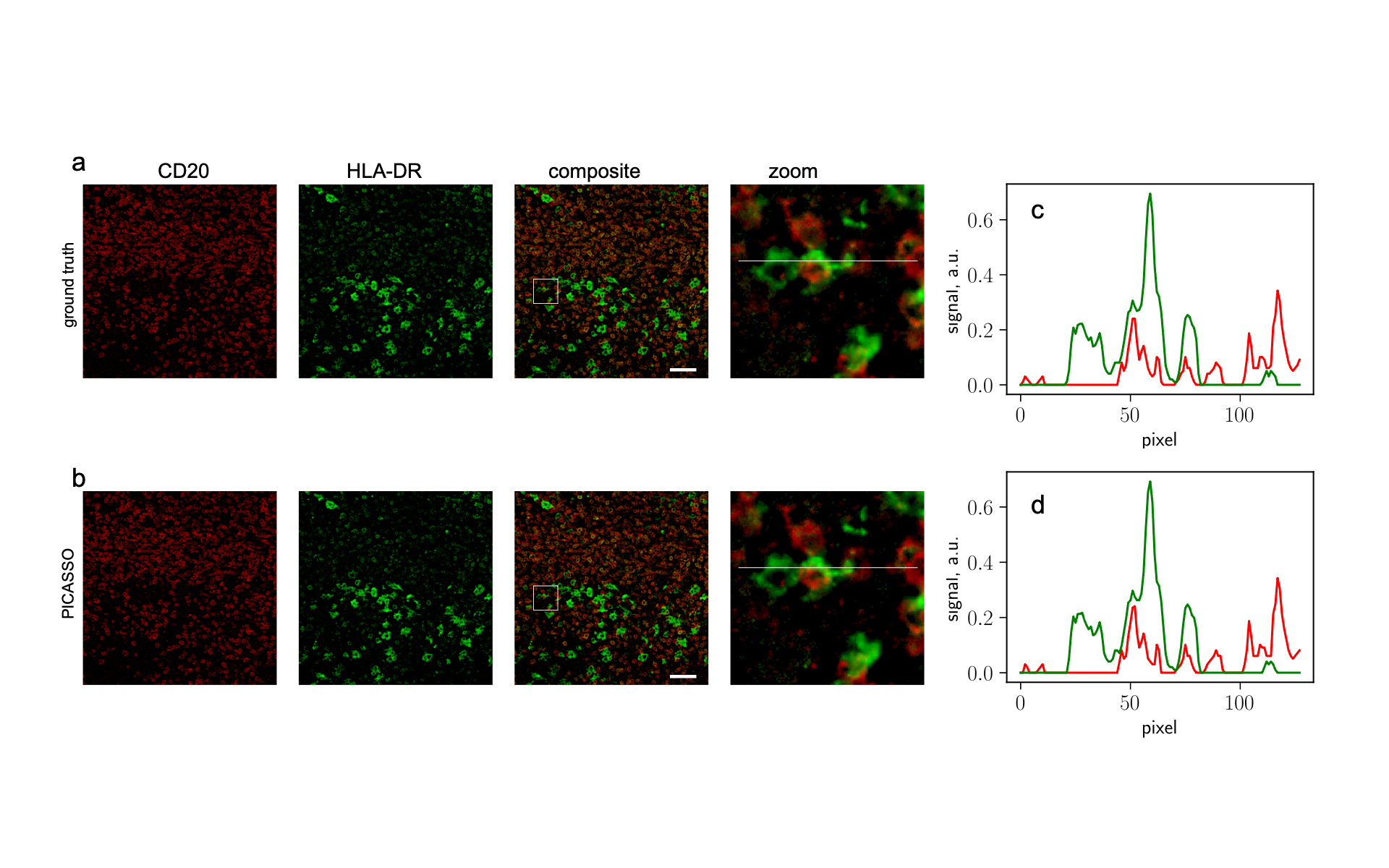

Supplement: btad784_Supplementary_Data [file btad784_supplementary_data.zip › sup_fig_1.png]

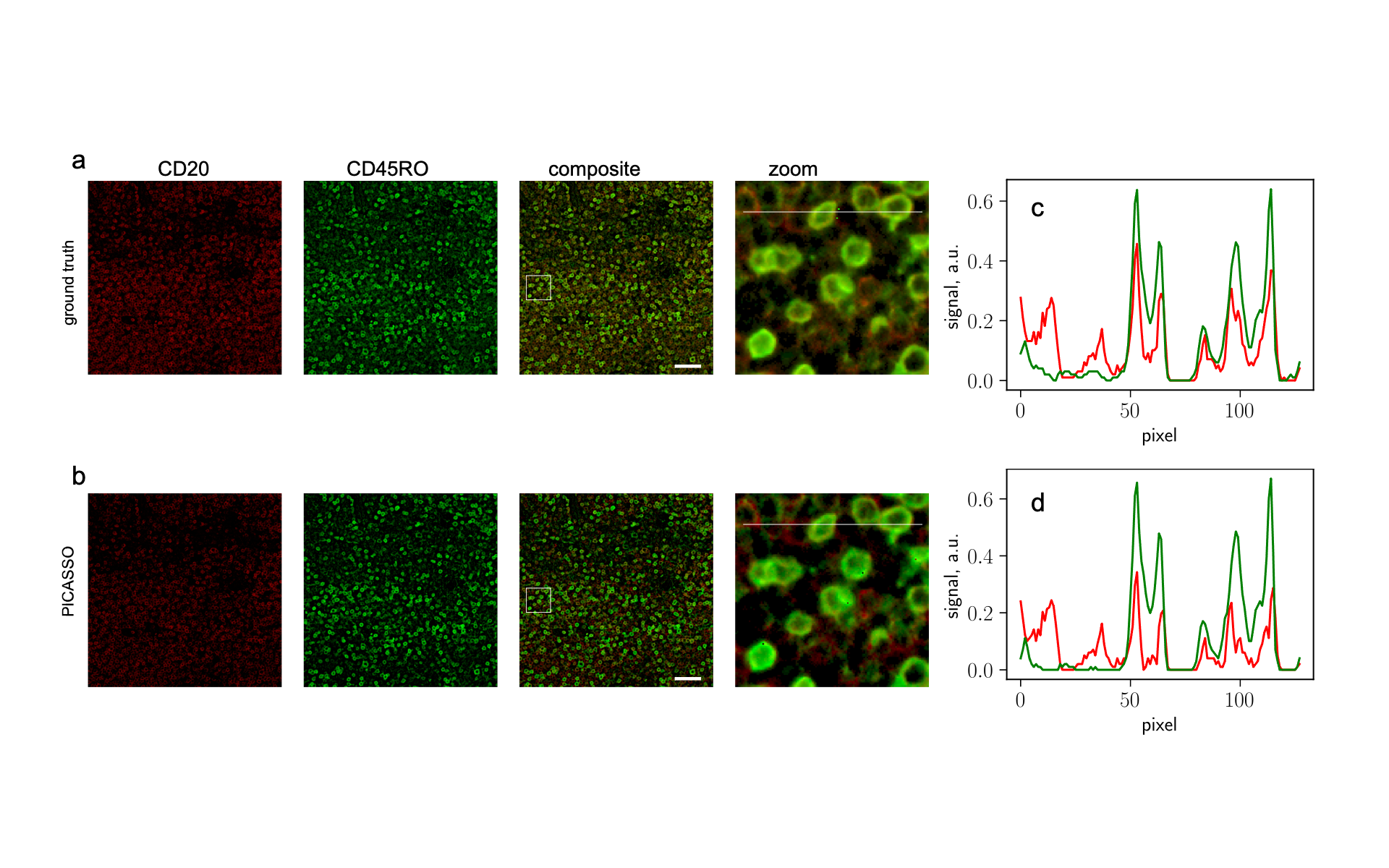

Supplement: btad784_Supplementary_Data [file btad784_supplementary_data.zip › sup_fig_2.png]
